# Cecal Perforation Secondary to Large Bowel Obstruction From a Tubo-Ovarian Abscess

**DOI:** 10.7759/cureus.29170

**Published:** 2022-09-14

**Authors:** Paige J DeBlieux, Thomas Herron

**Affiliations:** 1 Department of Acute Care and Trauma Surgery, Tampa General Hospital, University of South Florida Morsani College of Medicine, Tampa, USA

**Keywords:** obstetrics and gynecology, critically ill patients, cecal perforation, tubo-ovarian mass, acute care surgery and trauma

## Abstract

With the continued specialization of medicine, we as physicians often fall into the trap of placing pathologies into silos, focusing on what we are most practiced in caring for. When managing acute patients, it is important that we consider complications that can arise across systems and specialties which could place our patients at increased risk for morbidity and mortality. Tubo-ovarian abscesses (TOAs) are complex infections often arising in the setting of pelvic inflammatory disease. The resultant reactive inflammation is frequently the culprit of potentially fatal sequelae. This article looks to highlight a case of TOA that resulted in inflammation and obstruction of the adjacent large bowel which subsequently led to large bowel obstructions (LBOs) and perforation. Although LBO management is well described in the literature, perforation secondary to inflammatory compression from a TOA is rarely documented.

We present the case of a middle-aged female with significant comorbid conditions and recent prolonged retention of a tampon which likely acted as the nidus for the infection that led to her presenting pathology and need for admission, a left-sided TOA measuring 8.1 × 4.7 × 3.4 cm. Consultation by obstetrics-gynecology and interventional radiology determined that admission for observation and intravenous antibiotics alone was appropriate. The patient’s hospital course was complicated by enlarging TOA with peri-colonic abscess and acute decompensation in the setting of LBO and cecal perforation. Emergency laparotomy and right hemicolectomy by the acute care surgical team were performed. Postoperative management was complicated by septic shock which prolonged her hospital stay. Following inpatient optimization of nutrition and management of comorbid conditions, the patient was able to make a full recovery.

In patients with suspected TOA, special consideration should be given to surrounding structures, and potentially fatal complications should be kept in the forefront of the primary team’s minds. This case report aims to urge physicians caring for patients with TOA to maintain a high level of suspicion and consider how the benefits of aggressive management may outweigh those of conservative options.

## Introduction

A tubo-ovarian abscess (TOA) is a complex infection typically arising in the setting of pelvic inflammatory disease [1]. Formation of these abscesses can lead to a reactive inflammatory response which has the potential to obstruct adjacent structures such as the ureters and small and large bowel, leading to potentially fatal sequelae [1,2]. Although the management of large bowel obstruction (LBO) is well documented, LBO secondary to TOA and its specific considerations are rarely described [3-5]. We present a case of LBO with cecal perforation secondary to inflammatory compression from a TOA.

## Case presentation

**Figure 1:**
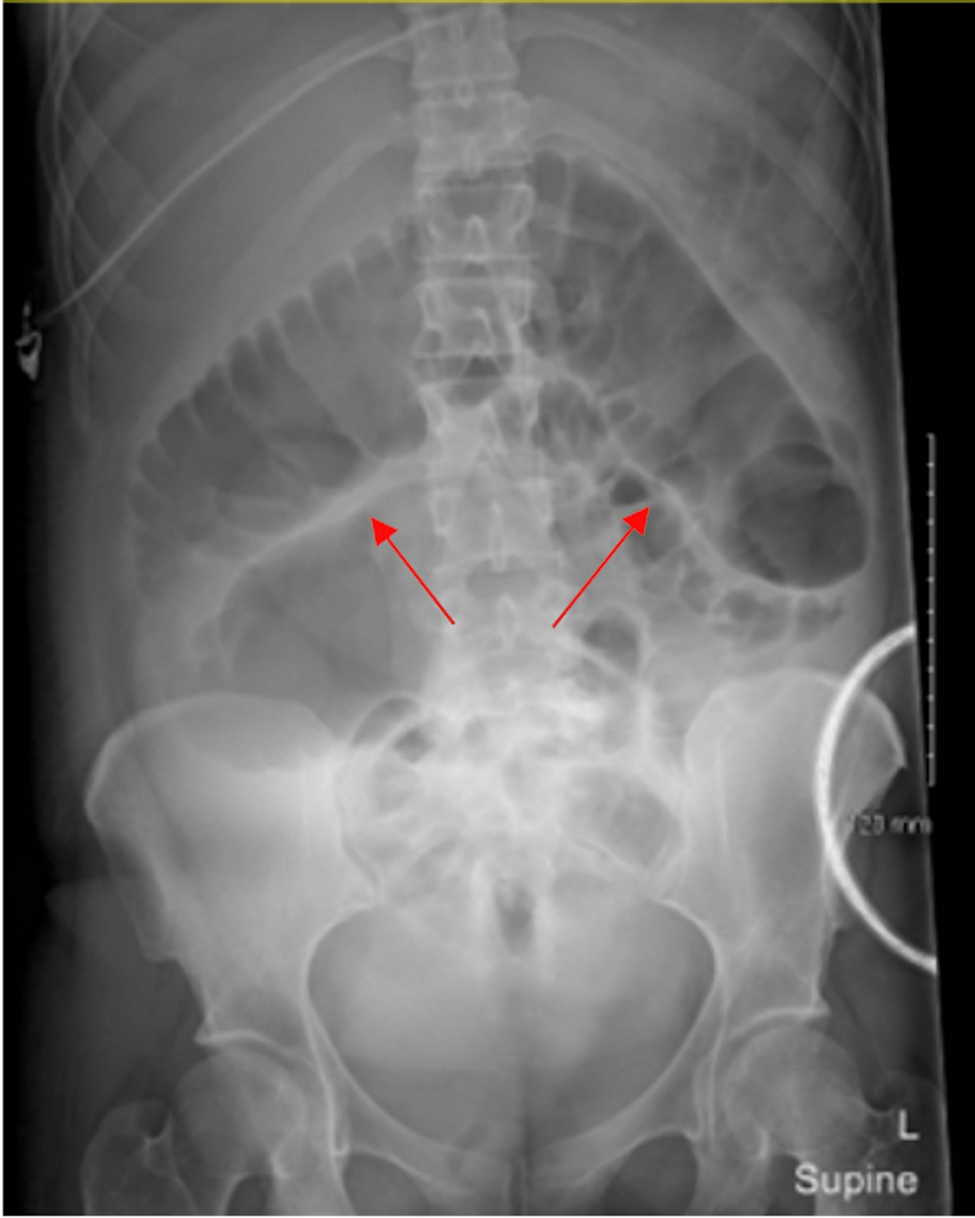
KUB demonstrating dilated loops of the bowel. KUB: X-ray of kidneys, ureters, and bladder

A 41-year-old female with alcoholic liver cirrhosis was admitted to our hospital following a diagnosis of left-sided TOA measuring 8.1 × 4.7 × 3.4 cm. During the initial interview, she disclosed recent prolonged retention of a tampon for one month that had been removed nine days prior to her current presentation. Upon admission, the obstetrics and gynecology (OB/GYN) team was consulted, and appropriate antibiotic therapy was initiated [6]. Following the initiation of antibiotic therapy, interventional radiology (IR) was consulted for potential drainage of the TOA. Given its containment within the fallopian tube, it was determined that drainage was not necessary. However, persistent leukocytosis one week after admission prompted further imaging. Results demonstrated an enlarging TOA measuring 9.2 × 6.7 × 3.8 cm and a possible new peri-colonic abscess. IR and colorectal surgery were subsequently consulted, and the enlarging TOA was managed with percutaneous drainage. Drain output totaled 298 ccs of purulent fluid, and antibiotic therapy was continued. Within 72 hours post-drain removal, the patient developed acute abdominal distention, early satiety, and failed to have a bowel movement. Successive X-rays of the kidney, ureter, and bladder demonstrated dilated loops of the bowel (Figure 1).

**Figure 2:**
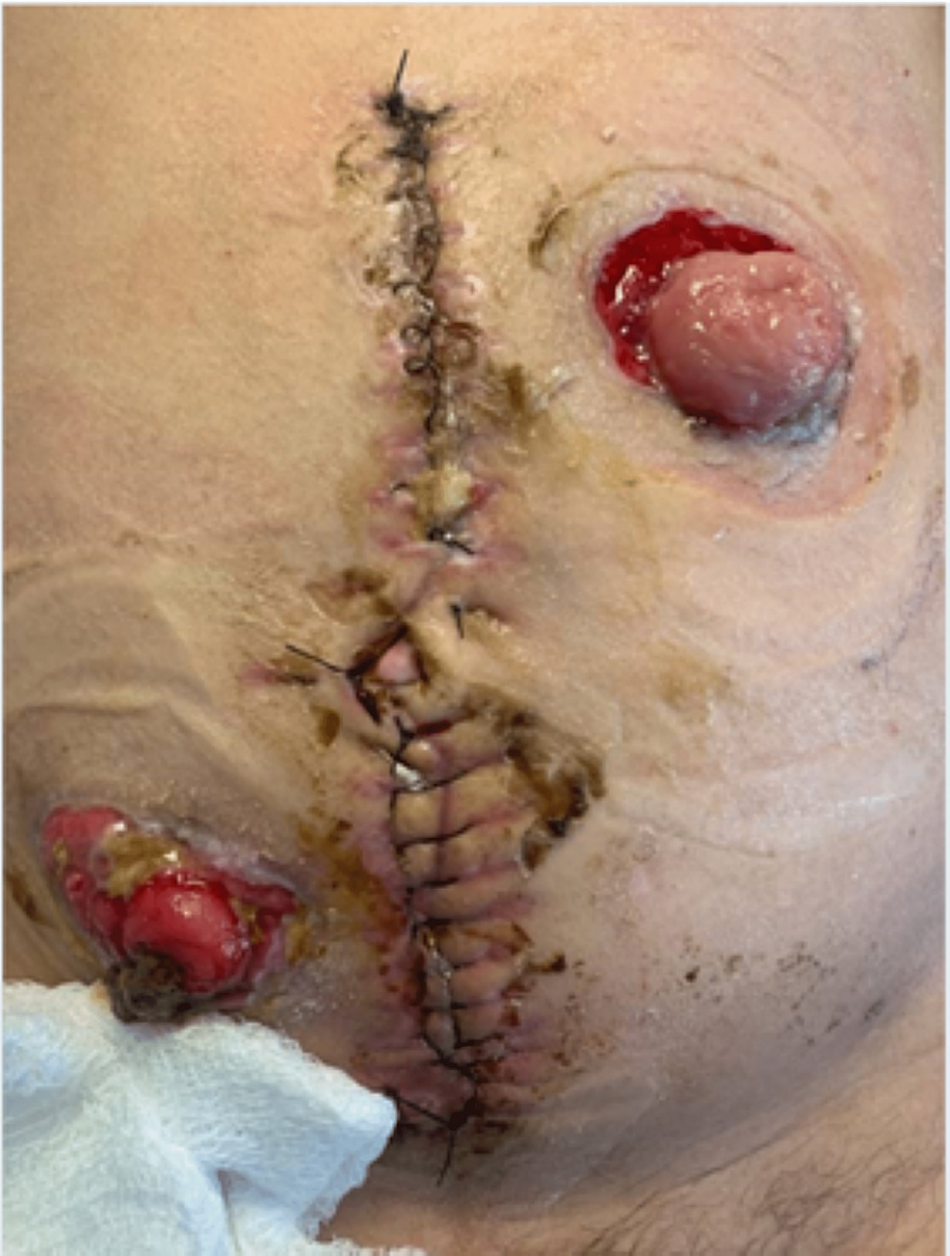
Postoperative end-ileostomy, transverse mucous fistula, and laparotomy incisional site.

The patient rapidly decompensated and developed septic shock while in the intensive care unit. It was at this time that the acute care surgery service was consulted for the management of an acute abdomen. Emergency laparotomy was performed, and upon entering the abdomen, 1.5 L of feculent ascitic fluid was encountered. After the evacuation of the fluid, the bowel was inspected and found to have multiple serosal tears, ischemia, and full-thickness perforations at the cecum. The small bowel was without damage, and the rest of the colon was dilated but viable. There was significant inflammation and adherence to the anterior-lateral wall at the sigmoid colon which was in proximity to the previous TOA. A decision was made to mobilize the right colon and establish a transverse colon mucous fistula for continued colonic decompression, rather than sigmoid resection, to give her a chance of ileostomy reversal in the future (Figure 2). Her immediate postoperative course was complicated by the need for acute management of septic shock, subacute management of ascites and malnutrition, as well as worsening liver function following stress from septic shock. After an extensive hospital stay of 34 days, the patient recovered and was discharged home. She recently had her end-ileostomy and mucous fistula reversed and continues working toward sobriety.

## Discussion

Sepsis occurs in 10-20% of patients who present with TOA, and mortality risk increases by 1.7-3.7% in those whose abscesses rupture [6]. Patients with abscesses ≥7 cm should undergo early surgical intervention with incision and drainage as well as pharmacologic treatment rather than antibiotic therapy alone [6,7]. In patients whose comorbidities may predispose them to worse outcomes if complications arise (i.e., poor glycemic control, immunodeficiency, poor nutritional status), a more aggressive initial approach may be appropriate. In addition, maintaining a high level of concern about the involvement of adjacent structures including the large and small bowels should be upheld throughout their care, even after initial management [8]. LBOs are surgical emergencies and require close observation of the cecum as its intrinsic characteristics make it particularly susceptible to perforation. The cecum is thin-walled and in close proximity to the ileocecal valve which impedes the retrograde flow of gas, increasing its risk of perforation, which is in accordance with the law of Laplace [9]. In this patient, the recent prolonged retention of a tampon likely created a nidus for infection which led to the formation of TOA. In the setting of poor nutritional status secondary to alcoholic liver cirrhosis and the TOA being >7 cm in diameter on presentation, this patient may have benefited from more aggressive initial management rather than conservative antibiotic therapy alone, despite containment within the fallopian tube. Lastly, it is always important when in a surgical emergency to stop and consider the long-term outcomes of perioperative decision-making. In this patient who is young and had a chance at recovery from alcohol use disorder, interim mucous fistula allowed her to undergo reversal and have some return of normalcy to her daily routine.

## Conclusions

TOAs measuring <7 cm may be managed with antibiotic therapy alone, while those measuring >7 cm should be approached more aggressively with incision and drainage in addition to antibiotic therapy. Given the high morbidity and mortality associated with complications of TOA, these patients should be monitored closely for signs of sepsis and obstruction of adjacent structures, even after initial therapy is provided. In patients suspected of having LBO, special consideration should be given to the cecum as early surgical intervention may prevent potentially fatal complications such as perforation and shock. Although distal LBO obstruction secondary to TOA is not well described, given the severity of possible complications, it is prudent that physicians maintain a high level of suspicion when caring for these patients.

## References

[1] Sam JW, Jacobs JE, Birnbaum BA (2002). Spectrum of CT findings in acute pyogenic pelvic inflammatory disease. Radiographics.

[2] Kairys N, Roepke C (2022). Tubo-Ovarian Abscess. https://www.ncbi.nlm.nih.gov/books/NBK448125/.

[3] Johnson WR, Hawkins AT (2021). Large bowel obstruction. Clin Colon Rectal Surg.

[4] Naveed M, Jamil LH, Fujii-Lau LL (2020). American Society for Gastrointestinal Endoscopy guideline on the role of endoscopy in the management of acute colonic pseudo-obstruction and colonic volvulus. Gastrointest Endosc.

[5] Lopez-Kostner F, Hool GR, Lavery IC (1997). Management and causes of acute large-bowel obstruction. Surg Clin North Am.

[6] Beigi RH (2022). Management and complications of tubo-ovarian abscess. UpToDate.

[7] Goje O, Markwei M, Kollikonda S, Chavan M, Soper DE (2021). Outcomes of minimally invasive management of tubo-ovarian abscess: a systematic review. J Minim Invasive Gynecol.

[8] Weledji EP, Elong F (2013). Small bowel obstruction and perforation attributed to tubo-ovarian abscess following 'D' and 'C'. World J Emerg Surg.

[9] Munro K, Gharaibeh A, Nagabushanam Nagabushanam (2018). Diagnosis and management of tubo-ovarian abscesses. Obstet Gynecol.

